# Personalized Monitoring and Advance Warning System for Cardiac Arrhythmias

**DOI:** 10.1038/s41598-017-09544-z

**Published:** 2017-08-24

**Authors:** Serkan Kiranyaz, Turker Ince, Moncef Gabbouj

**Affiliations:** 1 Department of Electrical Engineering, College of Engineering, Qatar University, Doha, Qatar; 2 Electrical & Electronics Engineering Department, Izmir University of Economics, Izmir, Turkey; 3 Department of Signal Processing, Tampere University of Technology, Tampere, Finland

## Abstract

Each year more than 7 million people die from cardiac arrhythmias. Yet no robust solution exists today to detect such heart anomalies right at the moment they occur. The purpose of this study was to design a personalized health monitoring system that can detect early occurrences of arrhythmias from an individual’s electrocardiogram (ECG) signal. We first modelled the common causes of arrhythmias in the signal domain as a degradation of normal ECG beats to abnormal beats. Using the degradation models, we performed abnormal beat synthesis which created potential abnormal beats from the average normal beat of the individual. Finally, a Convolutional Neural Network (CNN) was trained using real normal and synthesized abnormal beats. As a personalized classifier, the trained CNN can monitor ECG beats in real time for arrhythmia detection. Over 34 patients’ ECG records with a total of 63,341 ECG beats from the MIT-BIH arrhythmia benchmark database, we have shown that the probability of detecting one or more abnormal ECG beats among the first three occurrences is higher than 99.4% with a very low false-alarm rate.

## Introduction

**Figure 1:**
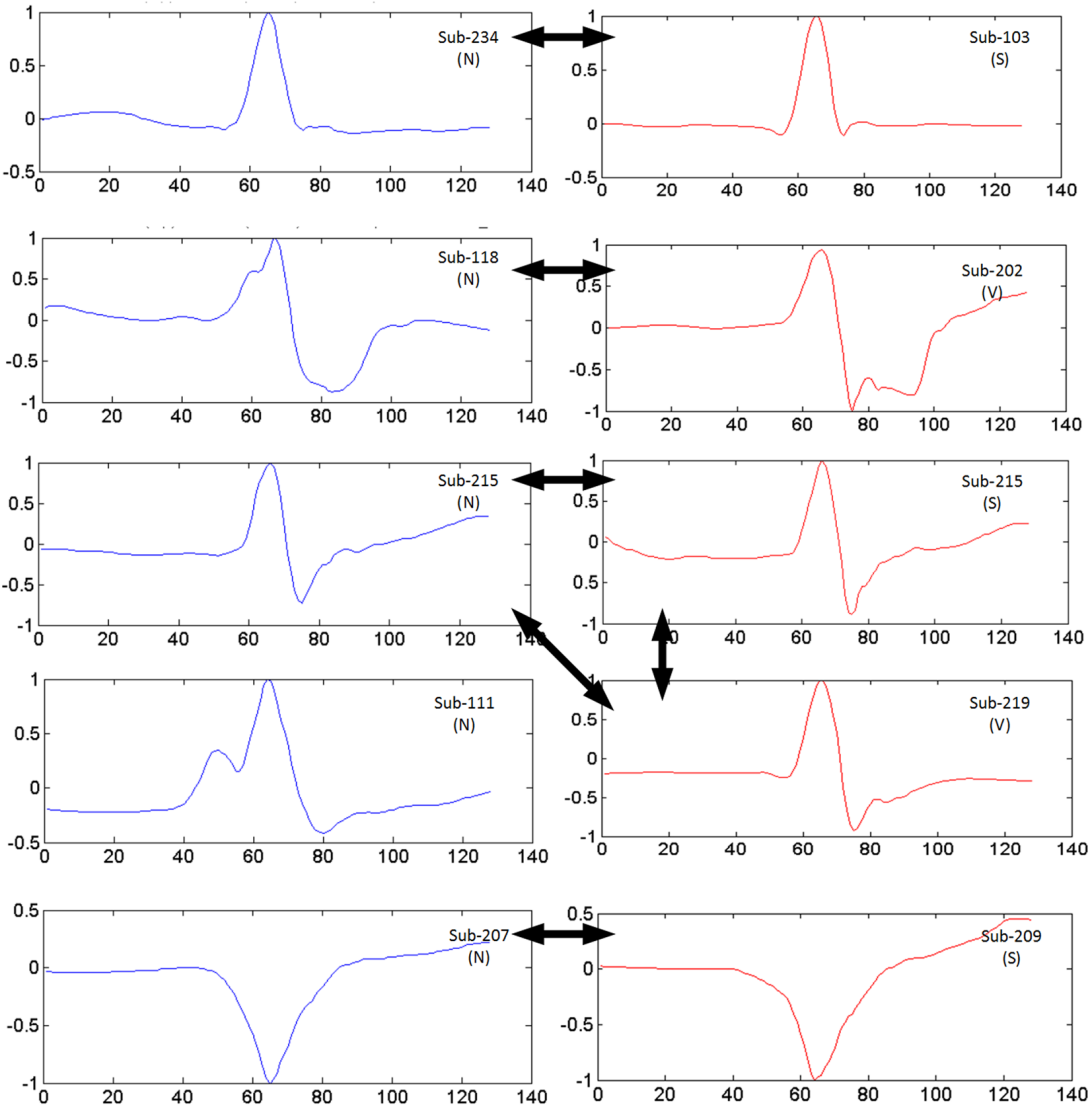
Normal (N) vs. Abnormal (S and V) beats from different subjects in MIT-BIH dataset.

Noninvasive diagnosis of arrhythmias is in general based on the standard 12-lead electrocardiogram (ECG), which involves measuring electric potentials from certain points on the body surface. Despite the fact that numerous methods have been proposed for generic ECG beat classification based on traditional signal processing and machine learning techniques, none has performed well in practice due to three major reasons: 1) High inter-patient variations of ECG signals which make a general purpose modeling and pattern learning infeasible, 2) Even for a healthy subject, QRS complex, P waves, and R-R intervals from one beat to another vary under different circumstances^1, 2^ and 3) the description capability of the extracted “hand-crafted” features which are often manually selected varies significantly among different ECG patterns. Figure 1 shows some typical ECG beats where different subjects in the benchmark MIT-BIH arrhythmia dataset^3^ have entirely different normal (N) beats, which may, however, show high level of similarities to other subject’s abnormal beats (e.g. see the pairs with arrows in the figure). As a result, the past approaches have usually exhibited a common drawback of having an inconsistent performance when, for instance, classifying a new patient’s ECG signal. This makes them unreliable for wide clinical use^4–6^. Such major deficiencies have recently led researchers to focus on *patient-specific* designs. Among them the studies in refs 4, 6–12 have demonstrated significant performance improvements over generic ECG classification methods thanks to their ability to adapt or optimize the classifier according to each patient’s ECG signal characteristics. Despite the success of these methods, they still require annotated samples (e.g. 5 minutes) containing both normal and abnormal beats of a patient for training or modeling. However, the ECG of a healthy person with no history of cardiac arrhythmias will exhibit no abnormal ECG beats. Therefore, none of them can be used as an advance warning system for cardiac arrhythmias.

**Figure 2:**
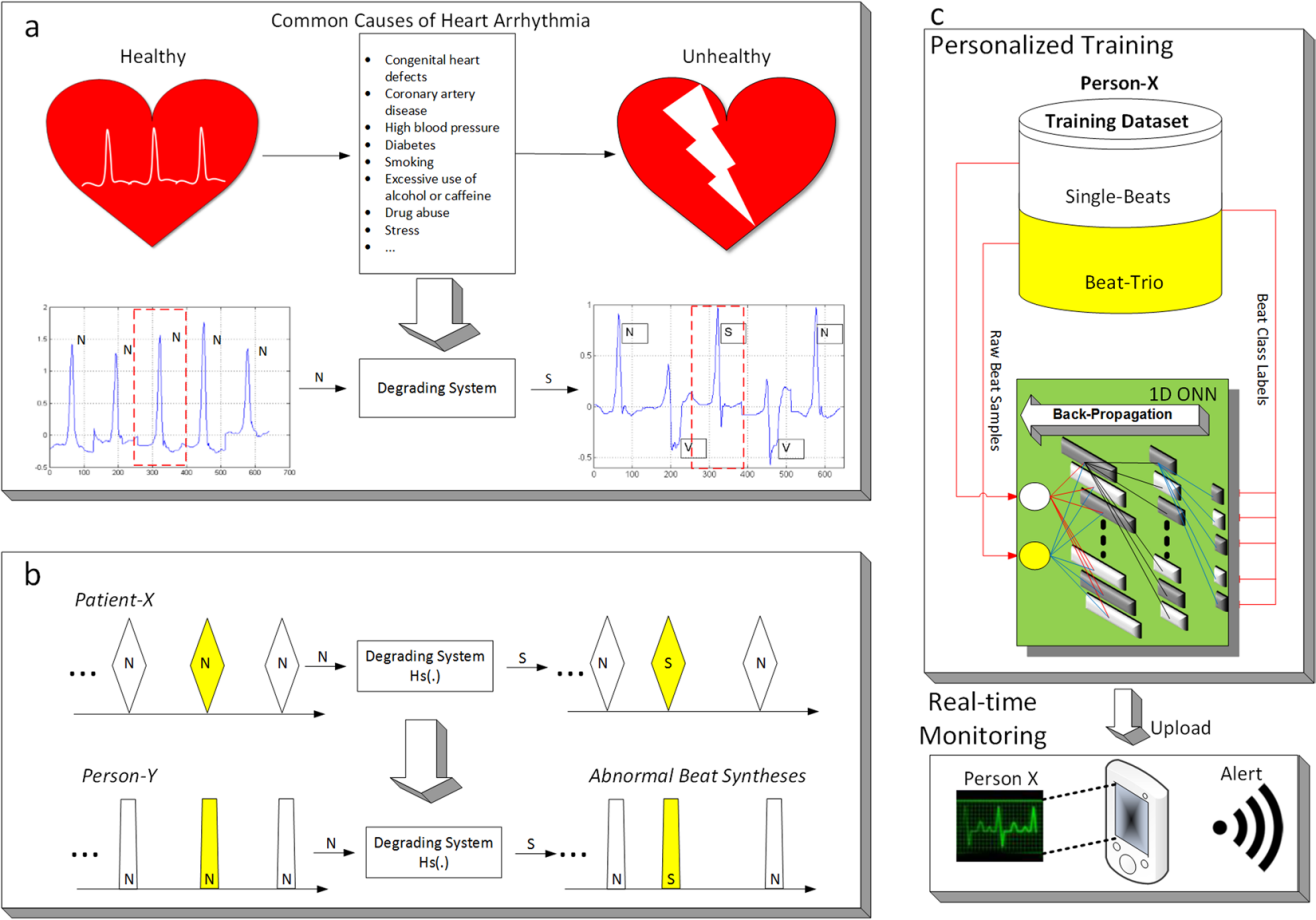
(**a**) Modeling common causes of cardiac arrhythmia in the signal domain by a degrading system. (**b**) A symbolic illustration of an abnormal *S* beat synthesis for *Person-Y* using the degrading system designed from the ECG data of *Patient-X*. (**c)** Illustration of the overall system, where a dedicated CNN is trained by Back-Propagation over the training dataset created for *Person-X* (top). Once the 1D CNN is trained, it can then be used as a continuous cardiac health monitoring and advance warning system (bottom) for *Person-X*.

In order to address this drawback efficiently and propose a reliable solution for the early detection of ECG anomalies, in this study we proposed an abnormal beat synthesis (ABS) approach, which can artificially create potential abnormal beats for a healthy individual using a library of filters applied to regular normal beats. To design such filters, we first modeled the common causes of arrhythmias such as smoking, high blood pressure, clotting, diabetes, drugs, etc., in the signal domain as a degradation of normal beats to abnormal beats. Using a benchmark dataset of ECG records, each normal-to-abnormal beat degradation was modeled by a distinct filter kernel. In this way, we aimed to model the common causes of heart health degradations that lead to abnormal beats, as a “degrading system” that turns regular normal beats to abnormal beats as shown in Fig. 2a. Our objective was not to clinically identify the underlying causes of the degradation, but only to model it with an ABS filter that can be used to produce a potential arrhythmic beat for a healthy person. Our main assumption was that if a healthy person will exhibit a similar arrhythmia in the future due to the same “common” cause (degradation), then the corresponding degrading system (the ABS filter) can synthesize those arrhythmic beats. Using the library of filters over an average normal beat, one can generate a set of potential abnormal ECG beats of that person and a dedicated classifier can thus be trained *in advance* to be used as an early detection system for cardiac arrhythmia. This approach is illustrated in Fig. 2b in a symbolic way where the common cause of a typical supraventricular premature ectopic (S) beat from *Patient-X* has been modeled by the linear filter kernel, Hs(.), which is then used to synthesize (a potential) S beat for *Person-Y*. In this way the classifier, 1D CNN, can learn in advance a potential S beat of *Person-Y* and can, henceforth, detect it as soon as it occurs.

The personalized ECG monitoring system presented in this study for the early detection of cardiac arrhythmias performs three prior and one-time (offline) operations: 1) Creation of the abnormal beat synthesis (ABS) filter library by performing regularized Least-Squares optimization (see Supplementary Methods), 2) Creation of the personalized training dataset via synthesis of potential abnormal beats using the ABS filter library over the person’s average normal beat (ANB), and 3) Training a dedicated Convolutional Neural Network (CNN) for that person over the training dataset created. The final step is illustrated in Fig. 2c over the personalized training dataset formed in the second step. The systematic approach of the proposed method is detailed in Methods section. The details of the first step and Back Propagation training method for CNNs are provided in the Supplementary Methods.

## Evaluation of ABS Filters

**Figure 3:**
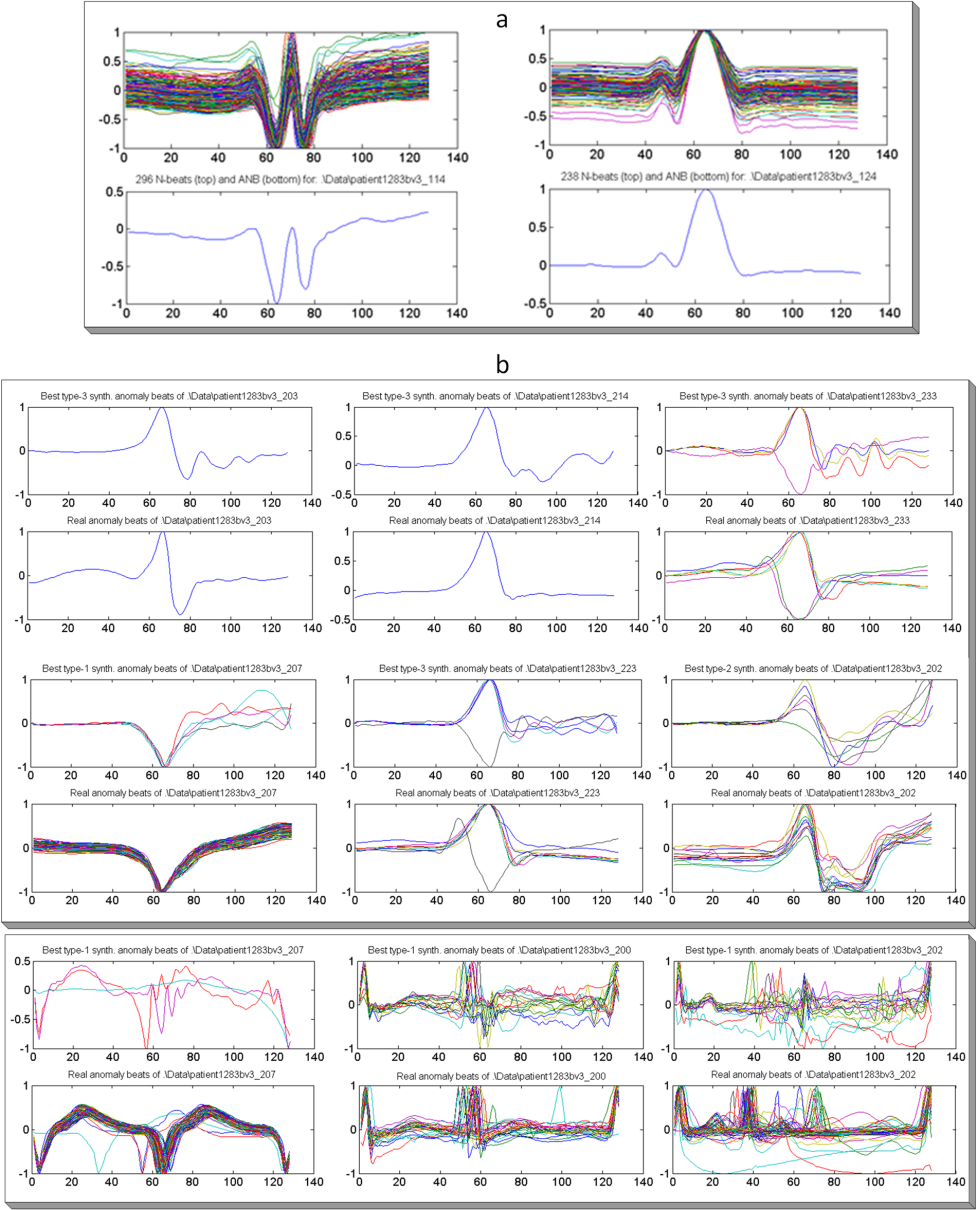
(**a**) Average normal beat (ANB) selections for Subjects 114 (left) and 124 (right). (**b**) Real beats and their most-similar synthesized beats of some subjects in the test partition. In both single-beat (upper block) and beat-trio (lower block) representations the bottom and top sub-plots show the real and the synthesized beats, respectively.

For the evaluation of Abnormal Beat Synthesis (ABS) we used the MIT/BIH arrhythmia database^3^. For both ECG beat representations (single-beat and beat-trio), first the average of the normal beats is computed using the first 5 minutes of the record. The normal N-beat that is closest to the average is selected as the average normal beat (ANB). Figure 3a shows two typical ANB selections for the two subjects with IDs 114 and 124.

Over the training partition of the database (subjects with IDs 1XX) we formed 464 filters (see Supplementary Methods for details) that comprise the ABS filter library. For each subject in the test partition, first the ANB was formed among the N-beats in the patient-specific part of the record. Then the abnormal beats were synthesized using the ABS filters in the library (excluding the ABS filters that are formed using the real abnormal beats of this subject) over the ANB of the subject. For the plots in this section we enumerated the following ECG beat types, N (normal), S (supraventricular ectopic), V (ventricular ectopic), F (fusion), and Q (unclassifiable) as follows: N = 0, S = 1, V = 2, Q = 3 and F = 4. For a visual evaluation, Fig. 3b shows real abnormal beats and the most similar abnormal beats synthesized by the ABS filters. Such typical results clearly demonstrate that the very similar abnormal beat patterns (S, V or Q) of each patient can indeed be synthesized with a proper ABS filter. Finally, for each subject in the test partition a personalized training dataset is generated (see Methods) over which a unique 1D CNN for that subject was trained.

## 1-D Convolutional Neural Networks

CNNs have recently become the *de-facto* standard for “deep learning” tasks such as object recognition in large image achieves as they achieved the state-of-the-art performance^14, 15^ with a significant performance gap. Typically, CNNs are feed-forward artificial neural networks (ANNs)^16, 17^. They have a structure with both alternating convolutional and subsampling layers, reminiscent of simple and complex cells in the human visual cortex^18–20^. As they primarily mimic the human visual system, which can efficiently recognize the patterns and structures (e.g. objects) in a visual scenery, CNNs are developed primarily for 2D signals such as images and video frames. However, recently 1D CNNs have successfully been used for the classification of electrocardiogram (ECG) beats^12^ achieving the state-of-the-art performance in terms of both accuracy and speed. The convolutional and sub-sampling layers basically process the raw data and learn to extract such features that can be used by the classification performed by the fully-connected layers. Therefore, both feature extraction and classification operations are fused into one body that is jointly optimized to maximize the classification performance. This is the main advantage of the adaptive 1D CNNs which can also yield such a low computational complexity that allows real-time implementation on a portable device.

## Abnormal Beat Detection Performance

As the main goal was the detection of the early occurrence of abnormal beats of an individual, some of the patients in the test partition were not suitable for the evaluation of the proposed method. Because the data comes from patients with severe heart conditions, it exhibits high variations among both normal and abnormal beats, and contains a large number of abnormal beats. Among 44 patients in the database, we have selected 34 subjects that show no or occasional cardiac arrhythmias and relatively low variations among the beats. The subjects with IDs, 105, 114, 201, 202, 207, 209, 213, 222, 223 and 234 were therefore, excluded. In particular, the four subjects with IDs, 115, 122, 212 and 231 have no abnormal beats and were thus ideal for evaluating the real False Alarm Rate (*FAR*) of the proposed method (0.11%). Overall, 34 patient records with a total of 63,341 ECG beats were selected for the evaluation.

Table 1 presents the Confusion Matrix (CM) cumulated by the classification results of 10 CNNs each of which is trained in a distinct backpropagation (BP) run. Table 2 shows the 2 × 2 deducted CM using the standard performance metrics (see Supplementary Methods Section 3): accuracy (*Acc*), sensitivity or recall (*Sen*), specificity (*Spe*), Positive Predictivity or Precision (*Ppr*) and false-alarm rate (*FAR*). We obtained: *Sen* = 82.51%, *Spe* = 99.55%, *Ppr* = 95.55%, *Acc* = 97.75% and *FAR* = 0.45%, respectively. The average probability of missing the first abnormal beat is 0.174 and the average probability of missing three consecutive abnormal beats is around 0.0053. Therefore, detecting one or more abnormal beat(s) among the first three occurrences is highly probable (>99.4%) by using the proposed method.

**Table 1:** Cumulated CM for the test dataset over 10 runs.

	Ground Truth					
Real		N	S	V	F	Q
	N	564118	1443	7447	2721	60
	S	1362	12913	9752	447	17
	V	856	217	28145	210	20
	F	224	16	1254	22	3
	Q	120	11	2032	0	0

**Table 2:** 2 × 2 confusion matrix deducted from the CM in Table 1.

	Ground Truth		
Real		N	A
	N	564118	11671
	A	2562	55059

## Discussion

In this study, we presented a real-time solution for the personalized health monitoring for the early detection of cardiac arrhythmias. In the absence of real abnormal beats, this task becomes a far more challenging problem compared to typical ECG beat classification systems for heart patients whose records exhibit both normal and abnormal beats. The final system was designed to detect any abnormal beats as soon as they occur with no prior training or tuning. To accomplish this objective, we first performed abnormal beat synthesis (ABS) for each subject using a library of ABS filters. Each filter in the library is optimized by the regularized Least-Squares as a model of a signal degradation corresponding to known physical causes of heart problems. Overall, the idea is to model the common causes of cardiac arrhythmias in an ABS filter library, which can then be used to synthesize potential abnormal beats of a healthy person.

Per AAMI ECAR-1987 recommended practice^13^, we performed an extensive set of experiments over the benchmark MIT-BIH arrhythmia dataset. Over the limited training data, the ABS filter library managed to synthesize such abnormal beats having similar patterns and characteristics compared to real abnormal beats. Once the 1D adaptive CNN is trained over the synthesized beats we achieved an exceptional cardiac arrhythmia detection accuracy especially over those ECG records that suit this objective. We further investigated the false-alarms for several patients in the test partition and realized that those normal ECG beats (classified as abnormal beats) indeed have a high level of temporal and morphological variations or degradations. Therefore, such occasional false alarms may still be useful as they may indicate some abrupt changes in the heart-beat morphology, pace, or both.

As a result, the proposed system achieved the main design objectives, i.e. maintaining a real-time, robust and personalized heart monitoring system for advance detection of cardiac arrhythmias. It is also a fully automatic and unsupervised system as it does not require any manual feedback or consultation from Cardiologists. To the best of our knowledge this is a pioneer work in *personalized* monitoring and advance warning system for cardiac arrhythmias. Future applications of this work will include studies to enrich the ABS filter library for a better arrhythmia modeling and test the proposed advance warning system on personal Holter registers.

## Methods

### ECG Data Processing

In this study, the benchmark MIT/BIH arrhythmia database^3^ was used for the synthesis of the ABS filter library and the performance evaluation of personalized abnormal beat detection. This benchmark database consists of 48 records, each containing of two-channel ECG signals of 30-min duration, each, selected from 24-hour recordings of 48 individuals. Continuous ECG signals are band-pass filtered within 0.1–100 Hz and then digitized at 360 Hz. The database contains annotation for both timing information and beat class information verified by independent experts. In the current study we used 44 records from the MIT/BIH arrhythmia database, that is, 4 records were excluded because they come from paced heartbeats. The first 20 records (with IDs in the range of 100 to 124), which include samples of routine clinical recordings, were used to select a number of representative beats to be included in the common training data. The remaining 24 records (with IDs in the range of 200 to 234) contain uncommon but clinically significant arrhythmias such as ventricular, junctional, and supraventricular arrhythmias^21^. These are commonly used as the testing partition of the database. AAMI recommends that each ECG beat be classified into the following five heartbeat types: N (beats originating in the sinus mode), S (supraventricular ectopic beats), V (ventricular ectopic beats), F (fusion beats), and Q (unclassifiable beats). For all records, we used the modified-lead II signals and the labels to locate beats in ECG data. The beat detection process is beyond the scope of this study, as many highly accurate (>99.5%) automated beat detection methods have been reported, i.e. refs 22 and 23.

The raw data of each beat was represented by 128 samples via down-sampling. There are two distinct beat representations: in the single beat representation, equal number of samples from each side from the R (center) point of the beat are used. In order to learn the temporal characteristics of each beat, a beat-trio is formed from its neighbor beats. Therefore, the difference in timing information of the center beat together with its neighbors in the beat-trio formation can indicate related ECG anomalies such as the presence of a supraventricular ectopic beat.

### Personalized Training Dataset Creation

Conforming to the AAMI recommendations, ABS filters were designed using the first 5 minutes of data plus the common training data selected among the records in the training partition of the MIT/BIH arrhythmia database. First the average normal beat (ANB) was selected among the N beats in the first 5 minutes. ANB was the sole input, *a*, of the ABS filters created from that subject. Then for each abnormal beat of the subject an M-length ABS filter was designed. Finally, two filter selection models were applied in order to eliminate similar and all-pass filters. The first model applies if abnormal beats of the subject are similar to each other. In this case, one or few representative filters will suffice to synthesize an abnormal beat from that patient. Whereas the second model applies when an abnormal beat is similar to the ANB. Especially for single beat representation, some S-beats can have the same pattern as an N-beat. Since our aim was to model the synthesis of abnormal beats from a normal beat, those “all-pass” filters can be left out. The most distinct filters, which yield the highest variances among the filter parameters were selected into the ABS filter library.

**Figure 4:**
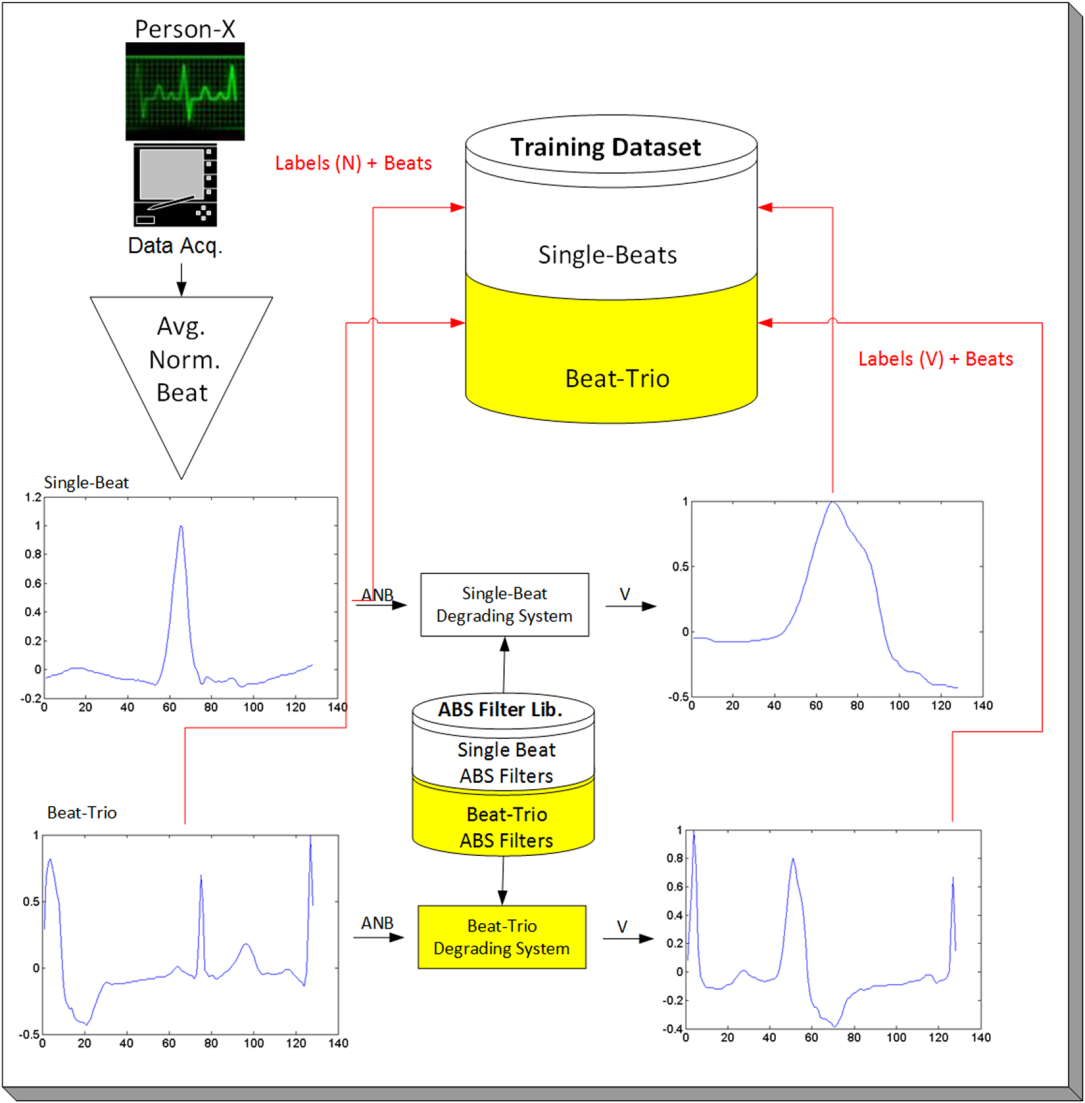
The creation of the training dataset for Person-X using a limited number of real N-beats.

For each beat representation, the ABS filter library was used to synthesize potential abnormal beats for the personalized training dataset of each patient in the test partition (subjects with IDs 2XX). The formation of a personalized training dataset is illustrated in Fig. 4. Since the proposed solution was intended for monitoring healthy subjects, no real abnormal beat was obviously used in the training dataset. In other words, the training dataset of each individual 1D CNN encapsulates only the *real* normal (N) beats of the subject in the test partition which are taken from the first 5 minutes of the record. Once trained with the real normal and the synthesized abnormal beats, the abnormal beat detection performance of the CNN was then evaluated over the real abnormal beats of the subject.

### Classifier Setup

In this study, we used a simple 1D CNN configuration with only 4 CNN and 2 fully-connected layers, in order to keep the CNN computationally efficient during the training and more importantly for real-time monitoring. The 1D CNN used in all experiments had 32, 16 and 16 neurons in the 1^st^, 2^nd^ and 3^rd^ hidden CNN layers, respectively, and 32 neurons in the hidden fully-connected layer. The output layer size was 5 which is the number of beat classes and the input (CNN) layer size was 2 so that both beat representations can be fed as inputs. The kernel size of the CNN was 7 and the sub-sampling factor is 3.

We employed a shallow training for the CNN: the maximum number of Back-Propagation (BP) training iterations is set to 50, and another stopping criterion, the minimum train classification error level, is set to 3% to prevent over-fitting. We initially set the learning factor, ε, as 0.001 and applied a global adaptation during each BP iteration: if the train MSE decreases in the current iteration we slightly increase ε by 5%; otherwise, we reduce it by 30%, for the next iteration. As BP is a deterministic gradient-descent optimization technique, which makes it quite dependent on the initial (random) setting of the network parameters, we performed 10 individual BP runs for each subject in the database and two detection performance metrics were reported, the average abnormal beat detection accuracy and the false alarm rate (see Supplementary Methods).

### The Overall System Workflow

In this study, we presented a real-time and fully automatic solution for personalized cardiac-health monitoring and early detection of cardiac arrhythmias from the electrocardiogram (ECG) data. The proposed solution involves a systematic approach to:

1. model the common causes of cardiac arrhythmias using a set of ABS filters.
2. synthesize all potential abnormal beats for a “healthy person” from his/her normal ECG beats.
3. form the personalized training dataset for the healthy person using the synthesized abnormal (arrhythmic) beats and real normal beats.
4. train the personal classifier, the 1D CNN, over the personalized training dataset.
5. use the personal classifier in real-time to detect any real arrhythmia –if and when occurs.

**Figure 5:**
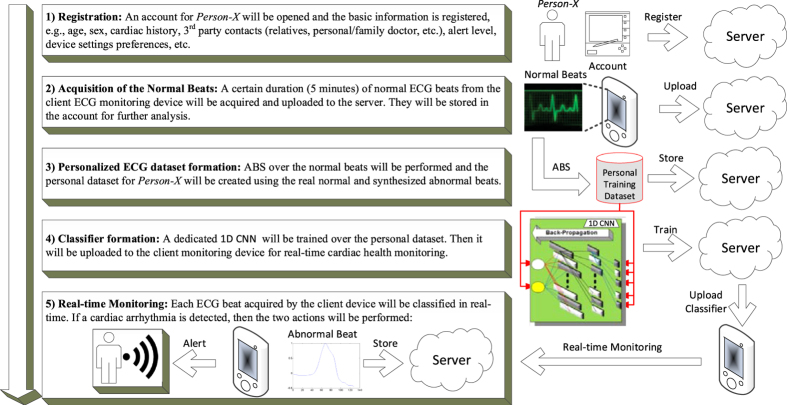
The system overflow of the proposed solution in an illustrative Client/Server application.

The overall flowchart of the proposed solution in an illustrative Client/Server application is shown in Fig. 5. In this application, all offline, one-time and computationally costly operations are executed at the server side, while the real-time monitoring and advance warning system is implemented at the client side, that is a wearable device. If the client detects any arrhythmia, then it will alert the user and store the arrhythmic beat(s) in the server for future medical verification and approval. In such an application, the server can further extract many other useful information such as arrhythmia types, statistics, frequency, time-of-occurrence, etc., to assist the Cardiologist in providing fast and reliable diagnosis.

## Electronic supplementary material

Supplementary Methods
