# Supplementary material for: Personalized Monitoring and Advance Warning System for Cardiac Arrhythmias
Source: Sci Rep. 2017 Aug 24;7:9270. doi: 10.1038/s41598-017-09544-z (PMC5571226; doi:10.1038/s41598-017-09544-z)
Supplement: Supplementary file 1 — Supplementary Methods [file 41598_2017_9544_MOESM1_ESM.pdf]

Supplementary Methods for:

Personalized Monitoring and Advance Warning System for Cardiac Arrhythmias

Serkan Kiranyaz, Turker Ince, and Moncef Gabbouj

The data analysis consists of the following methods:

1. **ABS Filter Design**
2. **ABS Filter Selection**
3. **1D Convolutional Neural Networks**
4. **Performance Evaluation Metrics**

1. ABS Filter Design

An abnormal beat synthesis (ABS) filter models the degradation transformation of a regular normal beat to become an abnormal beat. This degradation basically represents one of the common causes of the cardiac arrhythmias that physically degrades the healthy heart (regular normal beat) to an unhealthy one that produces abnormal beats. Our main assumption is that ABS filters are linear and time-invariant (LTI). With this assumption, one can write the input-output expression as,

$$\begin{aligned} &b[n] = h[n] * a[n] = IDFT\,(H(f).A(f)) \\ &a[n]: a[0], a[1], \ldots a[N-1] \\ &b[n]: b[0], b[1], \ldots b[N-1] \\ &h[n]: h[0], h[1], \ldots h[M-1] \end{aligned} \qquad (1)$$

where a and b are N-length input and output signals corresponding to (regular) normal and abnormal beats, respectively, and h is the M-length filter coefficients of the LTI system. $H(f)$ and $B(f)$ are the DFT of h and b, respectively. One can easily derive h from the IDFT of the ratio between the DFTs of b and a, as follows:

$$h[n] = IDFT\left(\frac{B(f)}{A(f)}\right) \qquad (2)$$

where A(f) and $B(f)$ are the DFT of the input and output signals a and b, *respectively*. However, the singularities and low values of $A(f)$ caused to noise will make it infeasible to compute h accurately. Instead, we shall derive h using the Least-Squares (LS) optimization directly. One can write the linear convolution as,

$$b[n] = h[n] * a[n] = \sum_{m=0}^{M-1} a[n-m]h[m] \quad (M \le N) \qquad (3)$$

Note that the convolution output will have the length, N+M-1 where we only consider the first N samples since the output signal (abnormal beat) has the same length as the input. This can be written in matrix equation as follows:

$$\begin{bmatrix} a[0] & 0 & 0 & 0 & \ldots & 0 \\ a[1] & a[0] & 0 & 0 & \ldots & 0 \\ a[2] & a[1] & a[0] & 0 & \ldots & 0 \\ \ldots & \ldots & \ldots & \ldots & \ldots & \ldots \\ a[N-1] & a[N-2] & a[N-3] & \ldots & \ldots & a[N-M] \end{bmatrix} \begin{bmatrix} h[0] \\ h[1] \\ \ldots \\ \ldots \\ h[M-1] \end{bmatrix} = \begin{bmatrix} b[0] \\ b[1] \\ \ldots \\ \ldots \\ b[N-1] \end{bmatrix} \qquad (4)$$

or equivalently in a linear system equation,

$$Ax = b \tag{5}$$

where A is the *NxM* matrix with the shifted input signal samples, $\boldsymbol{x}$ is the column vector of filter coefficients, (x(i) = h[i]) and $\boldsymbol{b}$ is the column vector of output signal, b. The LS solution of this equation, x_{LS}, can be expressed as follows:

$$x_{LS} = \min_{x \in R^M} \|b - Ax\|^2 = \left(A^T A\right)^{-1} A^T b \tag{6}$$

However, matrix, A may not be of full rank, i.e., *rank(* A *) = r < M*, in which case, $A^T A$ will be singular and the inverse cannot be computed. To address this problem, we make use of the Singular Value Decomposition (SVD) of A as follows,

$$A = U\Sigma V^T = \sum_{i=1}^{r} \sigma_i u_i v_i^T \tag{7}$$

where $\boldsymbol{U}$ and $\boldsymbol{V}$ are *NxN* and *MxM* orthogonal matrices which holds the eigenvectors of the square matrices, AA^T and $A^T A$, respectively, as the column vectors. The *NxM* matrix, Σ, can be expressed as,

$$\Sigma = \begin{bmatrix} \sigma_1 & 0 & 0 & \dots & 0 & 0 & \dots & 0 \\ 0 & \sigma_2 & 0 & \dots & 0 & 0 & \dots & 0 \\ 0 & 0 & \sigma_3 & \dots & 0 & 0 & \dots & 0 \\ \dots & \dots & \dots & \dots & \dots & \dots & \dots & 0 \\ 0 & 0 & 0 & \dots & \sigma_r & 0 & \dots & 0 \\ 0 & 0 & 0 & \dots & 0 & 0 & \dots & 0 \\ \dots & \dots & \dots & \dots & \dots & \dots & \dots & \dots \\ 0 & 0 & 0 & 0 & 0 & 0 & \dots & 0 \end{bmatrix} \tag{8}$$

where $\sigma_1 > \sigma_2 > ... > \sigma_r$ are the singular values or equivalently the eigenvalues of matrices, $A^T A$ and AA^T. This can yield the LS solution, x_{LS}, regardless whether or not A is singular,

$$x_{LS} = V\Sigma^{-1}U^T b = \sum_{i=1}^{r} \frac{1}{\sigma_i} v_i u_i^T b \tag{9}$$

However, the LS solution, x_{LS}, can still yield large values, the so-called "explosion" of the LS solution, due to noisy values in matrix A (the input) or in vector b (the output), or both since in general they are both susceptible to noise. A crucial disadvantage *therein* is that the smaller non-zero singular values will result in even larger explosion of x_{LS}. In order to prevent this, we shall *regularize* the LS solution by optimizing the LS error together with the magnitude of the LS solution as,

$$x_{RLS} = \min_{x \in R^M} \left(\|b - Ax\|^2 + \lambda^2 \|x\|^2\right) \tag{10}$$

where λ is the regularization parameter. It is straightforward to show that this joint optimization can be expressed as,

$$x_{RLS} = \min_{x \in R^M} \left\| \begin{pmatrix} b \\ 0 \end{pmatrix} - \begin{pmatrix} A \\ \lambda I \end{pmatrix} x \right\|^2 = \min_{x \in R^M} \|b_\lambda - A_\lambda x\|^2 \tag{11}$$

where A_λ is now an *Mx(M+N)* full-rank matrix (*r=M*) and therefore, the LS solution over A_λ can be obtained by using Eq. (6) as,

$$x_{LS}(\lambda) = \min_{x \in R^M} \|b_\lambda - A_\lambda x\|^2 = \left(A_\lambda{}^T A_\lambda\right)^{-1} A_\lambda{}^T b_\lambda \\ = \left(A^T A + \lambda^2 I\right)^{-1} A^T b \qquad (12)$$

The *i*th eigenvector of $\left(A_\lambda{}^T A_\lambda\right)$ can be obtained by solving,

$$A_\lambda{}^T A_\lambda v_i = \left(A^T A + \lambda^2 I\right) v_i = \left(\sigma_i^2 + \lambda^2\right) v_i \qquad (13)$$

So it is clear that matrix $A_\lambda{}^T A_\lambda$ has the same eigenvector, v_i, as matrix $A^T A$ but a larger eigenvalue, $\left(\sigma_i^2 + \lambda^2\right)$. Therefore, using the orthogonality of the eigenvectors, one can write the eigenvector decomposition of $A_\lambda{}^T A_\lambda$, and its inverse as follows:

$$A_\lambda{}^T A_\lambda = \sum_{j=1}^{M} \left(\sigma_j^2 + \lambda^2\right) v_j v_j^T = V \Lambda V^T \\ \left(A_\lambda{}^T A_\lambda\right)^{-1} = \sum_{j=1}^{M} \frac{1}{\left(\sigma_j^2 + \lambda^2\right)} v_j v_j^T = V \Lambda^{-1} V^T \qquad (14)$$

Using Eqs. (7) and (9) above yields the regularized LS solution, x_{RLS}, expressed as,

$$x_{RLS} = x_{LS}(\lambda) = \left(A_\lambda{}^T A_\lambda\right)^{-1} A^T b \\ = \left(\sum_{j=1}^{M} \frac{1}{\sigma_j^2 + \lambda^2} v_j v_j^T\right)\left(\sum_{i=1}^{r} \sigma_i v_i u_i^T\right) b \\ = \left(\sum_{j=1}^{r} \frac{\sigma_j}{\sigma_j^2 + \lambda^2} v_j u_j^T b\right) \qquad (15)$$

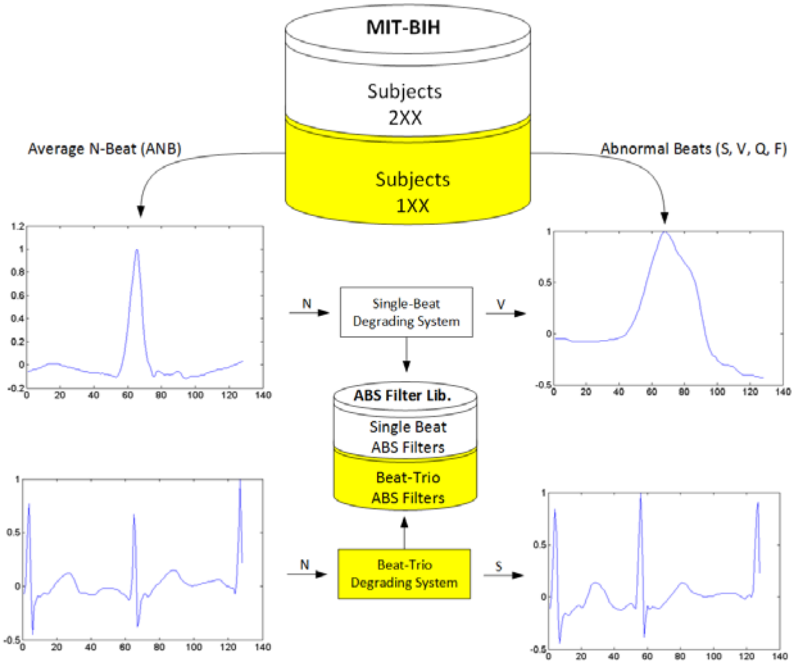

Figure 1: Illustration of two ABS filter designs: the top one is for a V-beat in single-beat representation and the bottom one is for an S-beat in a beat-trio representation.

Comparing regularized LS solution in Eq. (15) with the LS solution in Eq. (9), the explosive effects of those noise-like singular values over the solution can now be significantly suppressed with a practical choice of λ, e.g., $0.1 \le \lambda \le 0.5$. As a result, one can design an ABS filter using Eq. (15) for each pair of normal-abnormal ECG beats and as illustrated in **Figure 1**, a library of ABS filters can be designed for each representation (single-beat and beat-trio).

2. ABS Filter Selection

Comparing regularized LS solution in Eq. (15)with the LS solution in Eq. (9), the explosive effects of those noise-like singular values over the solution can now be significantly suppressed with a practical choice of , e.g., . As a result, one can design an ABS filter using Eq. (15) for each pair of normal-abnormal ECG beats and as illustrated in Figure 2, a library of ABS filters can be designed for each representation (single-beat and beat-trio). Finally, two filter selection models were applied to eliminate similar and all-pass (impulsive) filters. The former occurs if the abnormal beats of the subject are similar with each other. In that case one or few representative filters suffice to model abnormal beat syntheses from that patient. The latter occurs when the abnormal beat is similar to the ANB. Especially for single beat representation some S-beats can have the same pattern as an N-beat. Since the aim is to model the syntheses of abnormal beats from a normal beat, those "all-pass" filters can be left out. Both selections are performed by evaluating the mean-normalized variance of the filter coefficients. Those filters, which yield the highest variances will be selected into the ABS filter library. Over the training partition of the database we selected 464 filters to form the ABS filter library and used them to synthesize abnormal beats for the subjects in the test partition. We set $\lambda = 0.1$ and the minimum variances for filter selection are, 0.1 and 0.15 for the S and V type abnormalities, respectively. The reason for different variance settings is the scarcity of S beats compared to V beats, hence we balance the final number of filters for each of them. There is no selection for Q and F type anomalies since only a few (13+7=20) of them exists in the training partition. So, all ABS filters for Q and F type abnormal beats are kept in the library. Figure 2 presents the ABS filters formed for the V beats from the patient-specific training data of the Subjects with IDs 118 and 119. Note that for the Subject 119 only 4 out of 81 ABS filters are selected due to the high similarity among the filter coefficients while all filters are selected for the subject 118.

The reason we selected this many filters although there are only few dozens of common causes for arrhythmia is threefold: 1) It is desired to have more filters for a common cause which is more frequent among the arrhythmia patients. This will provide more samples for this type of arrhythmia and thus the personal classifier will have more positive samples for training. 2) This number of abnormal beats yields a balanced training dataset into which the normal beats from the first 5 minutes (i.e., 300-400 beats) are inserted. 3) Even multiple filters from the same degradation type (common cause) will emerge, the characteristics of the degradation occurred may be different and each filter can capture a distinct degradation instance. For example, the S type beat typically occurs with an early activation of the QRS waveform. However, the timing of the early activation may be different from subject to subject. Therefore, it makes sense to capture and model different S type occurrences with several ABS filters.

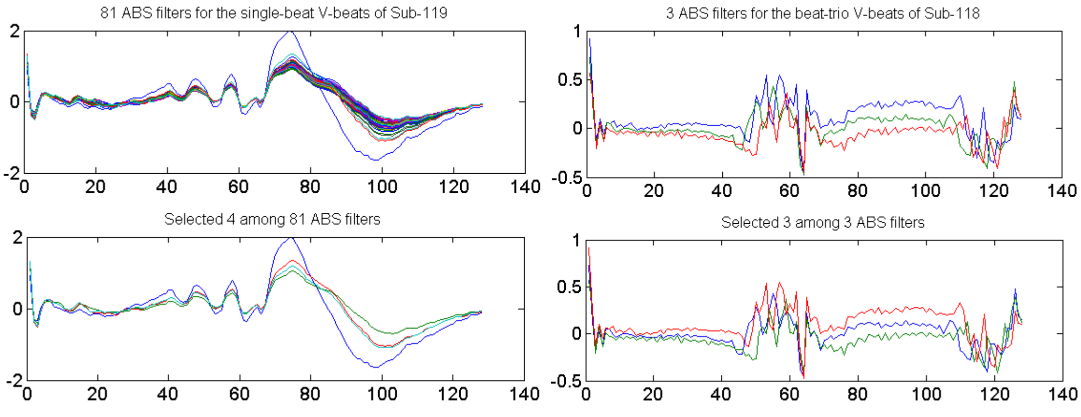

Figure 2: ABS filters (total and selected) for V beats for Subjects 119 (left) and 118 (right).

3. 1D Convolutional Neural Networks

In this study, we use the 1D Convolutional Neural Network (CNN) architecture shown in Figure 3. There are two

types of layers in the proposed 1D CNN: 1) CNN-layers where both 1D convolutions and sub-sampling occur, and 2) Fully-connected layers that are identical to the hidden and output layers of a typical Multi-Layer Perceptron (MLP).

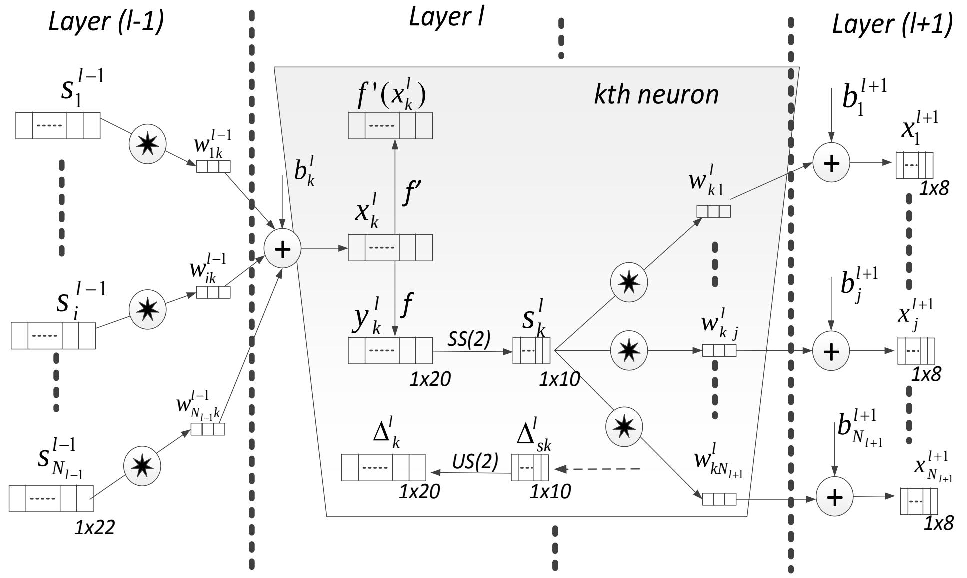

Figure 3: Three consecutive layers of the proposed 1D CNN architecture. The kth hidden neuron is exploited.

In the CNN-layers, the 1D forward propagation (FP) can be expressed as,

$$x_k^l = b_k^l + \sum_{i=1}^{N_{l-1}} conv1D(w_{ik}^{l-1}, s_i^{l-1}) \qquad (16)$$

where x_k^l is the input, b_k^l is the bias of the kth neuron at layer l, and s_i^{l-1} is the output of the ith neuron at layer l-1. w_{ik}^{l-1} is the kernel weight from the the ith neuron at layer l-1 to the kth neuron at layer l. With such an "adaptive" design it is aimed that the number of hidden CNN layers can be set to any practical number because the sub-sampling factor of the output CNN layer (the hidden CNN layer just before the first fully-connected layer) is set to the dimensions of its input map, e.g., in Figure 3 if the layer l+1 would be the output CNN layer, then the sub-sampling factors for that layer is automatically set to *ss* = 8 since the input map dimension is 8 in this sample illustration. Besides the sub-sampling, note that the dimension of the input maps will gradually decrease due to the convolution without zero padding, i.e., in Figure 3, the dimension of the neuron output is 22 at the layer l-1 that is reduced to 20 at layer l. As a result, the dimension of the input maps of the current layer is reduced by K-1 where K is the size of the kernel.

Back-Propagation Training for 1D CNNs

We shall now briefly formulate the back-propagation (BP) steps while skipping the detailed derivations due to the space limitations. The BP of the error starts from the output layer. Let l=1 and l=L be the input and output layers, respectively. The error in the output layer can be written as,

$$E = E(y_1^L, \ldots, y_{N_L}^L) = \sum_{i=1}^{N_L} \left(y_i^L - t_i\right)^2 \qquad (17)$$

For an input vector p, and its corresponding output vector, $[y_1^L, \ldots, y_{N_L}^L]$, we wish to compute the derivative of this error with respect to an individual weight (connected to that neuron, k) w_{ik}^{l-1}, and the bias of neuron k, b_k^l, so that we can apply gradient descent to minimize the error, accordingly. Once all delta errors in each fully-connected layer are determined by BP, the weights and bias of each neuron can be updated by the gradient descent method. Specifically, the delta of the kth neuron at layer l, Δ_k^l will be used to update the bias of that neuron and all weights of the neurons in the previous layer connected to that neuron, as:

$$\frac{\partial E}{\partial w_{ik}^{l-1}} = \Delta_k^l y_i^{l-1} \quad and \quad \frac{\partial E}{\partial b_k^l} = \Delta_k^l \qquad (18)$$

So from the input fully-connected layer to the output CNN layer, the regular (scalar) BP is simply performed as,

$$\frac{\partial E}{\partial s_k^l} = \Delta s_k^l = \sum_{i=1}^{N_{l+1}} \frac{\partial E}{\partial x_i^{l+1}} \frac{\partial x_i^{l+1}}{\partial s_k^l} = \sum_{i=1}^{N_{l+1}} \Delta_i^{l+1} w_{ki}^l \qquad (19)$$

Once the first BP is performed from the next layer, l+1, to the current layer, l, then we can further back-propagate it to the input delta, Δ_k^l. Let zero order up-sampled map be: $us_k^l = up(s_k^l)$, then one can write:

$$\Delta_k^l = \frac{\partial E}{\partial y_k^l} \frac{\partial y_k^l}{\partial x_k^l} = \frac{\partial E}{\partial us_k^l} \frac{\partial us_k^l}{\partial y_k^l} f^{'}(x_k^l) = up(\Delta s_k^l) \beta \, f^{'}(x_k^l) \qquad (20)$$

where $\beta = (ss)^{-1}$ since each element of s_k^l was obtained by averaging ss number of elements of the intermediate output, y_k^l. The inter-BP (among CNN layers) of the delta error ($\Delta s_k^l \xleftarrow{\Sigma} \Delta_\iota^{l+1}$) can be expressed as,

$$\Delta s_k^l = \sum_{i=1}^{N_{l+1}} conv\,1Dz\left(\Delta_\iota^{l+1},\, rev\,(w_{ki}^l)\right) \qquad (21)$$

where rev(.) reverses the array and $conv1Dz$(.,.) performs full convolution in 1D with K-1 zero padding. Finally, the weight and bias sensitivities can be expressed as,

$$\frac{\partial E}{\partial w_{ki}^l} = conv1D(s_k^l, \Delta_i^{l+1}) \quad \frac{\partial E}{\partial b_k^l} = \sum_n \Delta_k^l(n) \qquad (22)$$

As a result the iterative flow of the BP can be stated as follows:

1) Initialize weights (usually randomly, U(-a, a))
2) For each BP iteration DO:
   a. For each item (or a group of items or all items) in the dataset, DO:
      i. **FP**: Forward propagate from the input layer to the output layer to find the outputs of each neuron at each layer, $y_i^l, \forall i \in [1, N_l] and\ \forall l \in [1, L]$.
      ii. **BP**: Compute the delta error at the output layer and back-propagate it to the first hidden layer to compute the delta errors, $\Delta_k^l, \forall k \in [1, N_l] and \forall l \in [2, L-1]$.
      iii. **PP**: Post-process to compute the weight and bias sensitivities using Eq. (22)
      iv. **Update**: Update the weights and biases with the (cumulation of) the sensitivities found in (c) scaled by the learning factor, ε:

$$w_{ik}^{l-1}(t+1) = w_{ik}^{l-1}(t) - \varepsilon \frac{\partial E}{\partial w_{ik}^{l-1}}$$
$$b_k^l(t+1) = b_k^l(t) - \varepsilon \frac{\partial E}{\partial b_k^l} \qquad (23)$$

4. Performance Evaluation Metrics

As mentioned earlier, abnormal beat detection is a binary classification problem that assigns beats to either normal (N) or abnormal (S, V, Q, or F). Since this is originally a 5-class problem in order to compute the two binary detection performance metrics, abnormal beat detection accuracy (Acc) and false alarm rate (FAR), we shall first cumulate the 5x5 confusion matrix (CM) of each run and then deduce a binary (2x2) CM from the cumulated 5x5

CM. Let the cumulated CM be parametrized as in Table I where the ground-truth numbers are shown in the columns. The deduction of this 5*x*5 CM to 2*x*2 CM is basically the fusion of the abnormal classes (S, V, F and Q) into a single abnormal class (A), while keeping the normal (N) as the other class. The deducted CM is shown in Table II. The number true-negatives, (*TN*) is the number of N beats correctly classified which will be equivalent to *N0* in 5x5 CM. The number of false-positives, *FP,* is the number of misclassified N beats; therefore, $FP = N1 + N2 + N3 + N4$. The number of false-negatives, *FN,* is the misclassified abnormal beats, or equivalently the total number of abnormal beats classified as N beats. Therefore, $FN = S1 + V1 + F1 + Q1$. Finally, the number of true-positives, *TP* is the number of correctly classified (detected) abnormal beats and *TP* is, therefore, the sum of all entries in the red block in Table I. Note that we are ignoring the misclassifications among the abnormal classes as long as an abnormal beat is assigned to an abnormal class. With these definitions, we can define the standard performance metrics as follows: *Accuracy* is the ratio of the number of correctly classified patterns to the total number of patterns classified, $Acc = (TP+TN)/(TP+TN+FP+FN)$; *Sensitivity* is the rate of correctly classified events among all events, $Sen = TP/(TP+FN)$; *Specificity* is the rate of correctly classified nonevents among all nonevents, $Spe = TN/(TN+FP)$; and *Positive Predictivity* is the rate of correctly classified events in all detected events, $Ppr = TP/(TP+FP)$. Since there is a large variation in the number of beats from different classes in the training/testing data, *Sensitivity*, *Specificity*, and *Positive Predictivity* are more relevant performance criteria for medical diagnosis applications. Especially *Sensitivity* can also be interpreted as the average probability of detecting an abnormal beat accurately. Obviously, 1-*Sen* is also the probability of missing the detection of an abnormal beat. Therefore, the probability of missing all *n* consecutive abnormal beats will be, $P_n = (1-Sen)^n$. Finally, 1- P_n , will, therefore, be the detection probability of at least one or more abnormal beat(s) among *n* consecutive occurrences.

Table I: Parametrized 5x5 confusion matrix.

		Truth				
		N	S	V	F	Q
**Real**	N	***N0***	*S1*	*V1*	*F1*	*Q1*
	S	*N1*	***S0***	*V2*	*F2*	*Q2*
	V	*N2*	*S2*	***V0***	*F3*	*Q3*
	F	*N3*	*S3*	*V3*	***F0***	*Q4*
	Q	*N4*	*S4*	*V4*	*F4*	***Q0***

Table II: Parametrized 2x2 confusion matrix deducted from the CM in Table I.

		Truth	
		N	A
**Real**	N	***TN***	*FN*
	A	*FP*	***TP***
